# The Influence of the Depth of Grinding on the Condition of the Surface Layer of 20MnCr5 Steel Ground with the Minimum Quantity Lubrication (MQL) Method

**DOI:** 10.3390/ma15041336

**Published:** 2022-02-11

**Authors:** Wojciech Stachurski, Jacek Sawicki, Bartłomiej Januszewicz, Radosław Rosik

**Affiliations:** 1 Institute of Machine Tools and Production Engineering, Lodz University of Technology, Stefanowskiego 1/15, 90-537 Lodz, Poland; radoslaw.rosik@p.lodz.pl; 2 Institute of Materials Science and Engineering, Lodz University of Technology, Stefanowskiego 1/15, 90-537 Lodz, Poland; jacek.sawicki@p.lodz.pl (J.S.); bartlomiej.januszewicz@p.lodz.pl (B.J.)

**Keywords:** surface grinding, MQL, microhardness, residual stress, surface roughness

## Abstract

This paper describes the research on abrasive machining conditions and their influence on microhardness and residual stresses distribution in the technological surface layer of 20MnCr5 steel. The roughness of ground samples was also measured. Samples underwent a vacuum carburizing process (LPC) followed by high-pressure gas quenching (HPGQ) in a 4D quenching chamber. Processes were realized with a single-piece flow method. Then, the flat surfaces of samples were ground with a Vortex type IPA60EH20VTX alumina grinding wheel using a flat-surface grinder. The samples were ground to three depths of grinding (*a_e_* = 0.01; 0.02; 0.03 mm) with grinding fluid supply using either flood method (WET) or minimum quantity lubrication (MQL) method. The condition of the technological surface layer was described using microhardness and residual stresses, as well as some selected parameters of surface roughness. The results obtained revealed that changes in microhardness as compared to microhardness of the material before grinding were lower in samples ground with grinding fluid supplied with MQL method. At the same time, the values of residual stresses were also better for samples ground using MQL method. Furthermore, the use of grinding fluid fed with MQL method produced lower values of surface roughness compared to the parameters obtained with WET method. It was concluded that for the tested scope of machining conditions, the MQL method can be a favourable alternative to the flood method of supplying grinding fluid into the grinding zone.

## 1. Introduction

The grinding process can change surface layer properties such as fatigue strength, corrosion resistance and abrasion resistance [1,2]. Parameters like residual stress distribution, microhardness and surface roughness can determine the condition of the technological surface layer to a significant degree.

Microhardness and residual stresses are influenced, among other things, by the type of heat treatment before grinding as well as the properties of the grinding wheel, especially the type of abrasive material. The surface roughness obtained as a result of grinding is influenced by the active roughness of the grinding wheel corresponding to its topography and the specific volumetric productivity of grinding, which results from adopted setting values. Among these setting values, the grinding depth *a_e_* is of significant importance [3,4], just next to the workpiece speed *v_w_*.

The analysis of information on the types of heat treatment currently applied before grinding shows that carburising followed by quenching is one of the most commonly used methods of the surface heat treatment. Low-pressure carburising [5,6] is superior to conventional carburizing [7,8] in terms of efficiency and features a number of advantages, including lack of internal oxidation and higher uniformity of obtained layers. It should be remembered that residual stresses that have arisen as a result of thermochemical treatment (TCT) are present in the base and surface layers. The analysis of residual stresses is crucial due to their influence on, among others, fatigue strength, tribological wear, corrosion, brittle fracture and contact fatigue [9]. This influence may be beneficial, but it may also lead to the destruction of an element or the whole device, depending on the type of stresses and their superposition with operating stresses originating from external forces. The occurrence of compressive stresses in the surface layer compensated by tensile stresses in the core is believed [10,11] to contribute to the fatigue strength.

The grinding process that follows the thermochemical treatment is often carried out using grinding wheels with Al_2_O_3_ abrasive grains. Grinding wheels used in grinding of this type increase the grinding power resulting from the increased efficiency, which leads to temperature increment in the surface layer of the workpiece [1,3,4]. This is one of the reasons for changes in residual stresses and microhardness distribution in the material after heat treatment. Thermal loads cause both disadvantageous tensile stresses presence and microhardness changes in depth of the technological surface layer which reduces fatigue strength of dynamic loaded machine parts [2,12].

Furthermore, increasing grinding efficiency, e.g., by increasing the depth of grinding, also leads to the increased roughness of the machined surface. The size and shape of surface roughness influence on, among other things, the functional properties of the surface: abrasion resistance, suitability for transferring constant or variable loads, corrosion resistance, fatigue strength (decreases as the roughness increases). The deepest irregularities of roughness act in a notch-like manner, causing stresses concentration in locations of reduced cross-section and lowering them to the values at which micro-cracks are initiated. Corrosion foci are also formed in the recesses of the rough surface without the presence of variable loads [13]. It should be noted that reliable results can only be obtained on the basis of correctly measured and properly processed measurement data [14]. In addition, power spectral density (PSD) methods can be used for a detailed analysis of surface roughness [15].

With regard to the above, this article describes research works carried out on the influence of the depth of grinding as one of the important input parameters of the grinding process, which is crucial for its efficiency, on the condition of the technological surface layer 20MnCr5 alloy steel. This steel is designed for carburizing and is widely used in wind energy, automotive, aviation, and machinery. It is one of the most commonly used materials for the commercial production of transmission components (shafts, gears) that are subjected to high Hertz loads. In order to fully utilize the potential of this material, it is necessary to consciously shape the properties of its surface layer in both machining and abrasive machining. The authors of the work [16] point the need to ensure appropriate cutting conditions, giving special emphasis to the proper selection of cutting parameters and the type of tool material and their impact on the durability of the cutting tool during gear hobbing. In another study devoted to gear hobbing, the purposefulness of using the minimum GF flow rate supply by the MQL method, was demonstrated [17].

MQL (Minimum Quantity Lubrication) method involves the continuous generation of oil mist and feeding it directly into the grinding zone, usually onto the active surface of the grinding wheel. The most commonly used lubricating media are synthetic esters or fatty alcohols. For some time, mainly for ecological reasons [18,19], vegetable oils have also been used as a lubricating medium in the MQL method [20,21]. The flow of the lubricating medium is assisted by a transferring medium—a stream of compressed air [22,23], which, to a small extent, also acts as a cooling agent [24]. Published data indicate that in the MQL method, the lubricant is supplied in quantities of 10–500 mL/h [25,26,27]. In comparison, water-oil emulsions in the flood method are used in amounts exceeding 120,000 mL/h, whereas while grinding, the amount ranges from 300,000 to 1,200,000 mL/h, depending on the process technology.

It should be noted that the addition of Cr and Mn to the 20MnCr5 steel increases the hardenability, ensuring appropriate strength properties of the core and reducing the hardening stresses in the carburized layer. This makes abrasive machining of this steel difficult and requires appropriate conditions for the machining process. The works to date in this area [28,29] were devoted to the determination of the effect of the type of abrasive grain used on the microhardness and residual stress when applying GF by the flooding method and the MQL method. The conclusions presented in these papers show that the MQL method may, under certain processing conditions, be an alternative to the flood method. These conclusions are also confirmed by other studies [30,31], which show that the MQL method can improve the effectiveness of the GF reaching the contact zone between active abrasive grains and the ground surface and thus reducing the risk of an unfavorable thermal impact on the top layer.

The review of available literature has shown that the application of the MQL method during grinding is diversely evaluated in terms of the condition of the technological surface layer of the machined materials [32,33]. This is clearly understandable as the types of the grinding process and the conditions under which they are carried out may differ significantly [34,35,36]. Thus, it is not possible to directly implement the results of the research on the application of the MQL method done for a specific type of grinding process into the other types of grinding processes and to generalise them.

Accordingly, experimental tests, described in this article, were carried out with regard to the application of MQL method at different grinding depths *a_e_* when grinding flat samples made of 20MnCr5 steel (820 ± 10 HV). It should be expected that the use of the MQL method can provide machining conditions comparable or better to those provided by the flood (WET) method. Therefore, the purpose of this research was to determine the influence of selected abrasive machining conditions on the value and distribution of microhardness and residual stresses formed in the technological surface layer, as well as on the surface roughness after machining. The samples were vacuum carburised (LPC) first, using a single-piece flow method; then, high-pressure gas quenched (HPGQ), and then ground with a Norton Vortex-type alumina grinding wheel. During the grinding process, conventional GF was delivered with the WET method and with the MQL method. Section 2 presents the experimental test conditions along with a description of the test stands. Test results and their analysis are discussed in Section 3, while Section 4 provides the final conclusions.

## 2. Experimental Tests

### 2.1. Vacuum Carburising with Single-Piece Flow Method and Heat Treatment

**Figure 1:**
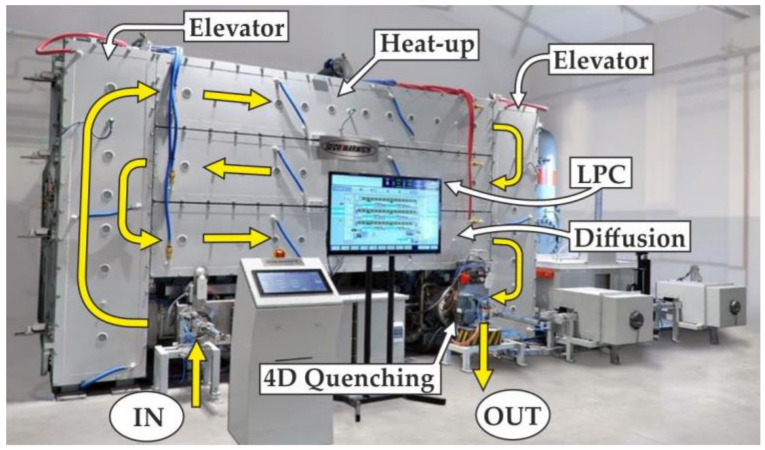
General view of UCM furnace (SECO/WARWICK) for low-pressure carburising.

The innovative vacuum UCM furnace made by SECO/WARWICK (Poland) shown in Figure 1 was used for the thermochemical treatment.

The special feature distinguishing this furnace from other vacuum furnaces is that the thermochemical treatment is carried out using the “single-piece flow” method rather than the batch method used so far. The furnace′s innovative design incorporates three process chambers (heat-up, LPC, diffusion) connected in parallel with each other, with individual workpieces moving in a flow. These chambers are configured in a horizontal layout and placed in a common vacuum space with gas tight separation. Transportation chambers (elevator) equipped with loading and unloading systems to cooperate with individual process chambers are built-in at the ends of these chambers. External access to transportation chambers is ensured by loading and unloading locks. The “single piece flow method” is that individual workpiece passes through similar process conditions and positions in the furnace [37,38]. The method provides high precision and repeatability in comparison to conventional carburizing methods. The 4D quenching chamber for individual high-pressure gas quenching (HPGQ) of single element allows controlling the cooling curve and achieving optimum properties of treated materials. A rotating table (4D) with the workpiece placed on together with application of uniformly distributed around cooling nozzles ensure even flow of gas and cooling ratios comparable to oil systems without the need of applying helium gas as cooling media.

Ring-shaped flat samples with an outer diameter of 96 mm, inner diameter of 30 mm and thickness of 10 mm were selected for experimental testing. The samples’ dimensions resulted from the construction of the elements of the mechanism that transports them inside the UCM furnace. Samples were carburised at 920 °C reaching the effective thickness of a layer ECD = 0.4 mm. Next, the samples were quenched in a quenching chamber at 7 bar and then tempered at 190 °C for 3 h. The parameters of the thermochemical treatment are presented in Table 1.

### 2.2. Grinding

The purpose of experimental testing of grinding flat surfaces was to determine the influence of changes in the grinding conditions, such as the depth of grinding and the method of GF supply, on the resultant process parameters in terms of microhardness, residual stresses and roughness of the ground surfaces.

**Figure 2:**
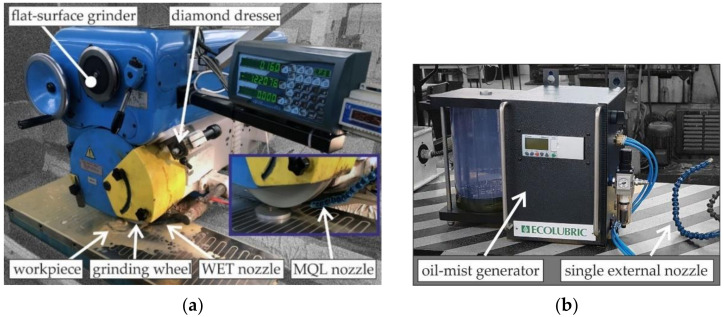
The experimental test stand with (**a**) an SPD-30B flat-surface grinder (Jotes Inc., Lodz, Poland) and (**b**) ecolubric MQL Booster applicator (Accu-Svenska AB, Västerås, Sweden).

Grinding was carried out on samples made of 20MnCr5 steel (820 ± 10 HV) that had previously been submitted to the thermochemical treatment process described in the previous subchapter. Table 2 shows the chemical compositions of 20MnCr5 steel. Grinding tests were carried out during the process of circumferential grinding of flat surfaces using a conventional flat-surface grinder of SPD-30B type (Jotes Inc., Lodz, Poland). View of test stand is presented in Figure 2a.

During the test the Vortex type grinding wheel made of alumina abrasive grains and ceramic binder—IPA60EH20VTX (Norton Saint-Gobain Ltd., Koło, Poland) were used. It is a hard grinding wheel with an open structure and increased porosity (so-called large-pore grinding wheel). The grinding wheel was dressed prior to each grinding test, using a single grain diamond dresser type M1020. Table 3 shows the grinding conditions employed during the experimental tests.

Grinding parameters used in tests are typical parameters used during circumferential grinding of flat surfaces. Machining allowance was removed in a single pass (concurrent direction), applying three grinding depths: *a_e_*_1_ = 0.01 mm, *a_e_*_2_ = 0.02 mm and *a_e_*_3_ = 0.03 mm. A constant value of the circumferential speed of the grinding wheel *v_s_* = 30.2 m/s and the workpiece speed *v_w_* = 18 m/min were assumed for the tests.

The test samples were ground with GF supplied by the WET method and with the MQL method. A water-oil emulsion using Emulgol ES-12 oil (5%) was used as the conventional grinding fluid in the flood method and it was fed into the grinding zone through a single nozzle with a flow rate of *Q_WET_* = 4 L/min (Figure 2). An external Ecolubric MQL Booster device (Accu-Svenska AB, Västerås, Sweden) shown in Figure 2b [39] was used to generate oil mist in the MQL method. During the tests, a single spray nozzle positioned tangentially to the active surface of the grinding wheel was used and GF was delivered at a flow rate of *Q_MQL_* = 100 mL/h (Figure 2a). Ecolubric E200L rapeseed oil, supplied by the machine manufacturer [40], was applied as the grinding fluid in the MQL method. Table 4 presents the technical characteristics of this oil.

Table 5 shows the set of variable machining conditions applied in the tests described above.

### 2.3. Microhardness Measurement

**Figure 3:**
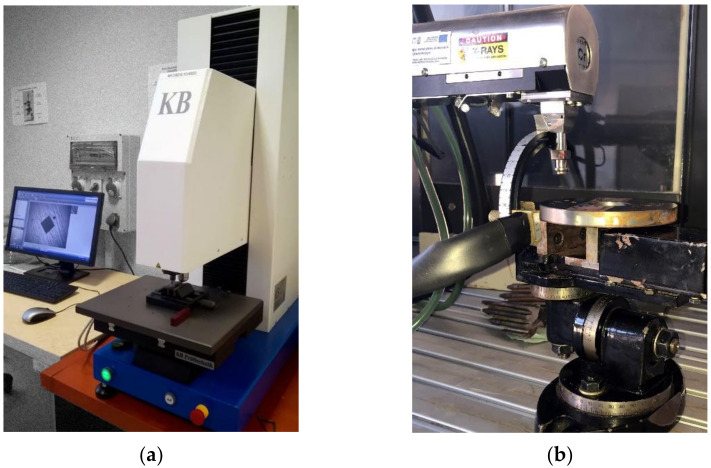
Measurement stands. (**a**) KB10BVZ-FA microhardness tester (KB Prüftechnik GmBH, Hochdorf-Assenheim, Germany); (**b**) PROTO iXRD X-ray diffractometer (Proto Manufacturing Ltd., LaSalle, ON, Canada).

Microhardness of the ground samples was measured with KB10BVZ-FA microhardness tester (KB Prüftechnik GmbH, Hochdorf-Assenheim, Germany), presented in Figure 3a. Microhardness distribution was assessed in accordance with PN-EN ISO 6570 at a load of 0.9807 N. Three microhardness distribution curves were collected on each sample to the depth 1 mm. The mean results obtained were interpolated using B-spline functions.

### 2.4. Residual Stress Measurement with Roentgen Method

Residual stress distributions were measured on the ground samples using PROTO iXRD apparatus (Figure 3b). An X-ray tube emitting characteristic Cr Kα radiation with wavelength *λ* = 2.29 A was used. Measurements were carried out in accordance to EN 15,305 standard using sin2ψ method in ω geometry. Changes in the position of iron (211) peak were recorded. X-ray diffractometric constants ½ S2 = 5.92 1/TPa and S1 = −1.27 TPa were taken for stress values calculation. The area measured was limited by a collimator 2 mm in diameter. The in-depth distribution of the residual stresses was obtained by successive electrochemical spot etching with the use of an 8818-V3 electropolisher delivered by PROTO.

### 2.5. Surface Roughness Measurement

**Figure 4:**
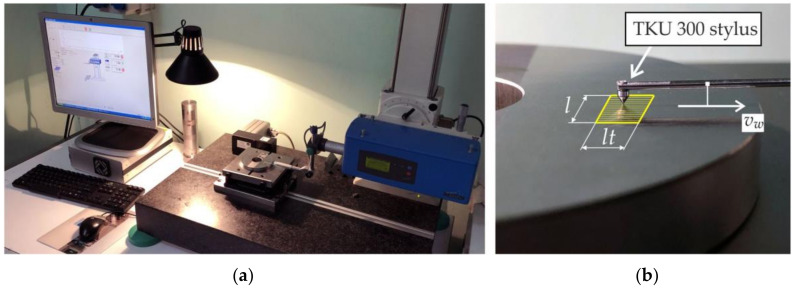
Surface roughness measurement. (**a**) General view of Hommel Tester T8000 profilometer; (**b**) Scheme of surface topography measurement.

Surface roughness measurements of the samples after grinding were made with a Hommel Tester T8000 profilometer (Hommelwerke GmbH, Schwenningen, Germany), which is presented in Figure 4a.

The measurement conditions were established in accordance with PN-EN ISO 3274:2011 and PN-EN ISO 4288:2011 and are presented in Table 6. 2D parameters, determined from the roughness profile, and 3D parameters, obtained as a result of surface topography measurements, were used to describe the roughness of the ground surface (Figure 4b). The following parameters were used as 2D parameters (according to PN-EN ISO 4287:1999/A1:2010): the value of the maximum height of the roughness profile *Rz*, the value of the highest peak of the profile *Rp* and the value of depth of the lowest valley of the profile *Rv*. The values of *R* parameters for each sample were calculated as the arithmetic mean of five measurements made on its ground surface. The amplitude parameters—*Sz*, *Ssk* and the parameters of the material ratio curve—*Sk* and *Spk* were used as 3D parameters (according to PN-EN ISO 25178:2019). It should be noted that the selection of roughness parameters was dictated by the requirements of the industrial project under which the tests were carried out. The literature provides information on the method of selecting parameters, e.g., parameters of the material ratio curve [41].

## 3. Results and Discussion

### 3.1. Microhardness

**Figure 5:**
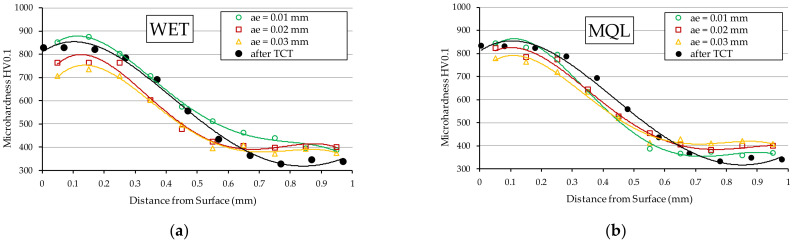
Microhardness distribution in 20MnCr5 steel ground with GF supplied with the following method: (**a**) WET; (**b**) MQL.

Microhardness tests carried out after grinding showed that the smallest changes (by about 7 to 50 HV near the surface) as compared to the microhardness of the material before grinding, are obtained when grinding with grinding fluid fed with MQL method (Figure 5b). Examination of the sample ground with grinding fluid supplied with WET method revealed microhardness reduction near the surface by approximately 65 to 120 HV (Figure 5a).

**Figure 6:**
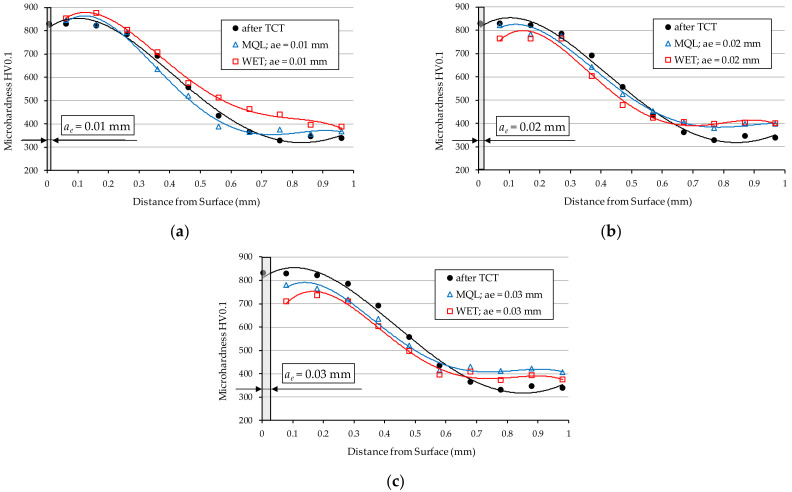
Microhardness distribution in 20MnCr5 steel ground with GF supplied with WET and MQL methods for grinding depth *a_e_*, which is: (**a**) 0.01 mm; (**b**) 0.02 mm; (**c**) 0.03 mm.

At the same time, as shown by the microhardness distributions presented in Figure 6, the greatest changes in microhardness values for both analysed grinding fluid feeding methods were observed for the grinding depth: *a_e3_* = 0.03 mm (Figure 6c). These changes were present starting from the surface down to the depth of 0.4 mm. In the case of grinding fluid fed with the MQL method, the reduction in microhardness ranged from 50 HV at the surface to 65 HV at a depth of 0.35 mm, as compared to the initial material. In the case of grinding fluid fed with the WET method, these values ranged from 120 HV to 90 HV, respectively.

Furthermore, the microhardness distribution obtained for the grinding depth *a_e_*_1_ = 0.01 mm and shown in Figure 6a, showed no significant differences compared to the material after thermochemical treatment. In case of depth *a_e_*_2_ = 0.02 (Figure 6b), the greatest changes in microhardness were observed on the surface of the sample ground using the WET method. The decrease in microhardness occurs up to a depth of 0.2 mm and is over 60 HV compared to the material after TCT.

### 3.2. Residual Stresses

**Figure 7:**
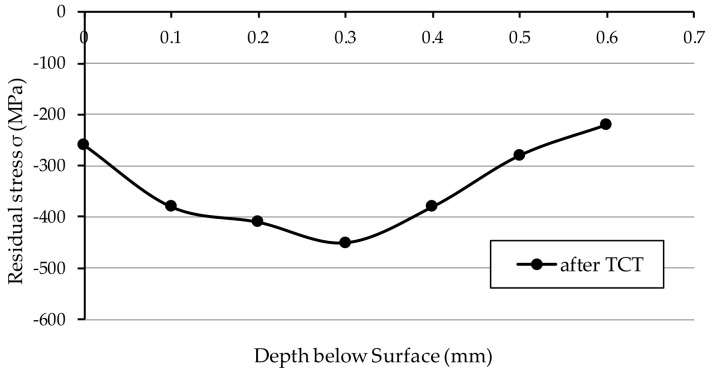
Residual stress in 20MnCr5 steel—after TCT, before grinding.

Figure 7 shows the results of residual stresses measurements presented as the mean value of the three measurements in samples after TCT, before grinding. The value of the residual stresses on the surface of the vacuum carburised samples was −260 MPa. Subsequently, as it can be seen from the graph, these stresses increased monotonically as the distance from the surface increased, reaching a value of −450 MPa at a depth of 0.3 mm, and then reaching a value of −220 MPa at a depth of 0.6 mm.

**Figure 8:**
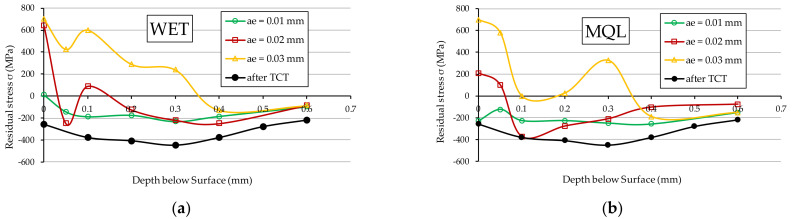
Residual stresses in 20MnCr5 steel ground with GF supplied with the following method: (**a**) WET; (**b**) MQL.

**Figure 9:**
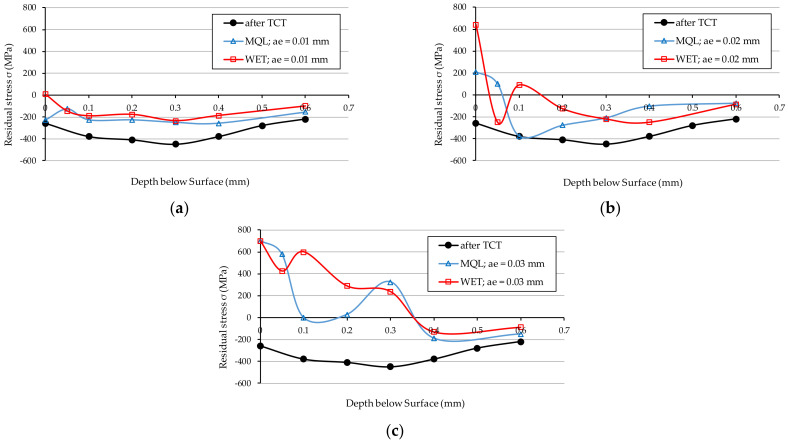
Residual stresses in 20MnCr5 steel after grinding to the *a_e_* depth: (**a**) 0.01 mm; (**b**) 0.02 mm; (**c**) 0.03 mm.

Figure 8 presents the stress distribution in the surface layer of vacuum carburised samples and then ground with a Vortex type IPA60EH20VTX grinding wheel using a grinding fluid supplied with the flood method—WET (Figure 8a) and with the MQL method (Figure 8b). Figure 9 shows the stress distribution for the same samples, at three *a_e_* grinding depths: 0.01 mm (Figure 9a), 0.02 mm (Figure 9b) and 0.03 mm (Figure 9c). A curve representing the residual stresses in the samples after TCT, before grinding, was also drawn in all the graphs.

As shown in Figure 8, grinding with the IPA60EH20VTX grinding wheel deteriorates the residual stress conditions compared to the material after thermochemical treatment (before grinding). This observation applies to the samples ground using the WET method and the MQL method.

For grinding depths *a_e_*_2_ = 0.02 mm (Figure 9b) and *a_e_*_3_ = 0.03 mm (Figure 9c), unfavourable tensile residual stresses were obtained just below the surface of the samples. This property applies to both methods of supplying the grinding fluid to the grinding zone. This is due to a large amount of heat transferred to the workpiece and the relatively high grinding temperatures causing unfavourable structural changes (among other things, tempering of steel). Grinding to a depth of *a_e_*_1_ = 0.01 mm (Figure 9a) using the GF supply with MQL method slightly changed the compressive stress values compared to the condition of the sample material after TCT. In contrast, the use of the WET method of supplying GF at this depth of grinding on the surface only resulted in the generation of low-value tensile stresses of 10 MPa, which at a depth of 0.05 mm transform into compressive stresses of −145 MPa and have a similar distribution pattern to those obtained using the MQL method.

As it can be seen in Figure 9a,b, more favourable values of residual stresses were obtained for samples ground with GF fed by the MQL method. This proves that the lubrication ability of this method is better than that of the WET method. In the authors’ opinion, the aforementioned property is a result of the porous structure of the Vortex-type IPA60EH20VTX grinding wheel featuring large inter-grain spaces and good penetration properties of such a structure by the oil sprayed at a high velocity through the nozzle of the oil mist generator in the MQL method. As a result, a large amount of lubricant is supplied to the contact zone between the active abrasive grains and the workpiece material, which reduces friction and leads to the reduction of the grinding temperature. As a consequence, lowering the friction ratio between the active abrasive grains and the workpiece surface leads to a lower grinding temperature, which has a great impact on the values of residual stresses occurring in the technological surface layer.

In the case of grinding at a depth of *a_e_*_3_ = 0.03 mm (Figure 9c), the GF supply method had no effect on the stress distribution, and in both cases (WET and MQL), significantly high tensile stresses were obtained, which have a very negative effect on the ground sample. This phenomenon is mainly due to the considerable depth of grinding, which creates material compaction, and, at the same time, no effective heat evacuation is ensured from the grinding zone.

The spikes visible on graph representing changes in residual stress values with depth are the result of synergistic interaction of the following processes: possible tempering and point re-hardening of the surface in contact with grinding wheel and cooling media, phase changes in the region of the heat-affected zone, namely tempering of existing martensite together with transforming retained austenite into martensite. In addition, the preexisting residual stresses state also takes part in the creation of the final measured values in general in a very unpredictable way. These lead to violently occurring changes of residual stresses state in depth of measured volume.

### 3.3. Surface Roughness

**Figure 10:**
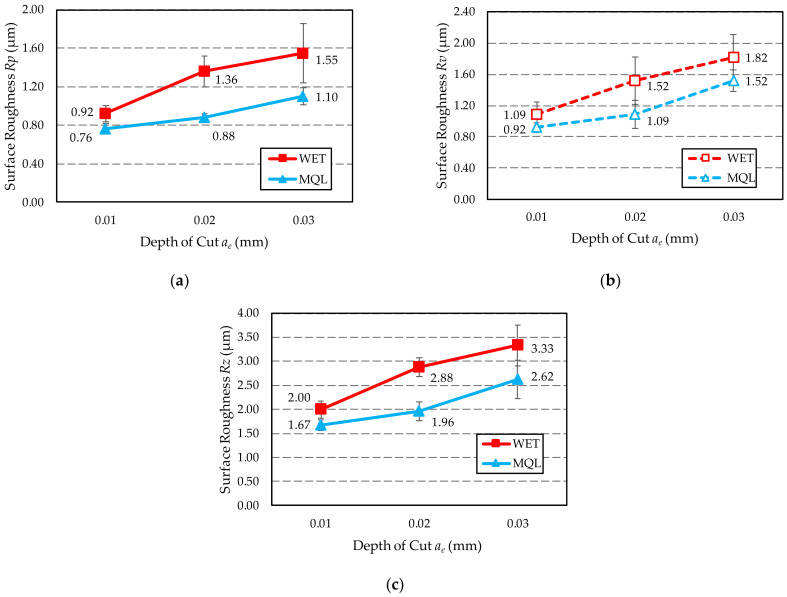
Value of 2D parameters determining the roughness of ground surface with WET and MQL methods: (**a**) *Rp*; (**b**) *Rv*; (**c**) *Rz*.

Table 7 compares the results of surface roughness measurements described by amplitude 2D parameters of the roughness profile—*Rp*, *Rv* and *Rz*. Figure 10 presents a graphical representation of the obtained results in the form of graphs.

The obtained results proved that for each of the three *a_e_* grinding depths, the lowest values of the *Rz* parameter (Figure 10c) were measured on the surface ground when Ecolubric E200L oil was fed with the MQL method. In the case of the water-oil emulsion fed with WET method, the obtained surface roughness is 20–47% higher compared to the surface roughness after grinding with GF fed using the MQL method. The gap between the compared roughness values depends on the depth of grinding. The lowest difference was obtained for the grinding depth *a_e_*_1_ = 0.01 mm.

The above observations indicate that within the range of the examined machining conditions, the GF feeding with MQL method ensures better lubrication in the contact zone between the tips of active abrasive grains and the workpiece, which translates into lower surface roughness. This may be explained by the fact that in this method, as a result of supplying the grinding fluid at a high speed to the grinding zone, oil particles can more effectively penetrate into free spaces between abrasive grains of the active surface of the grinding wheel. As a result, it ensures better friction conditions in the contact area of the grinding wheel with the ground surface compared to grinding using the flood method. Higher friction in the grinding zone in the case of the WET method, causes the high temperature in the surface layer of the workpiece to induce tempering of the surface, which is evidenced by the measured microhardness value (Figure 5c and Figure 6). In this type of surface, increased elastic deformation occurs during grain infeed, manifested by the formation of high lateral bumps and the occurrence of furrowing. This makes roughness increase in the ground surface. It should also be noted that when the surface is tempered, shavings adhere to it and cause surface abrasion, which also increases the roughness of the ground surface.

It is worth noting that the microhardness of the surface layer of the workpiece ground with a grinding depth *a_e_*_1_ = 0.01 mm using both methods of GF supply, did not significantly decrease compared to the microhardness of the material before grinding (Figure 6a). In view of the above, there were fewer elastic deformations in the shavings formation zone compared to the displacement occurring when higher grinding depths were used (*a_e_*_2_ = 0.02 mm and *a_e_*_3_ = 0.03 mm). Under these conditions, as the active abrasive grains move through the material, they form flakes of smaller height and a greater proportion of the volume of removed material is converted into shaving by micro-cutting. This results in lower values of the parameter *Rz* of the surface roughness.

**Figure 11:**
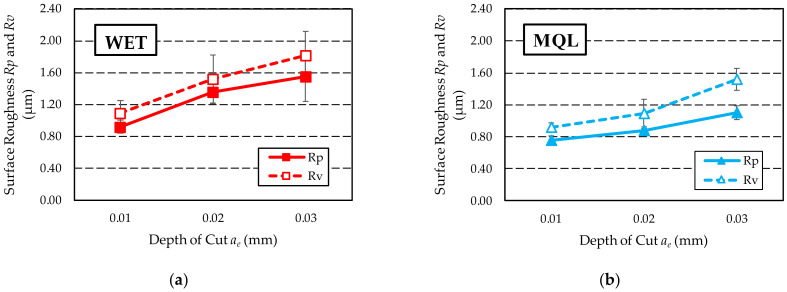
Comparison of surface roughness *Rp* and *Rv* parameters obtained with two methods of GF supply: (**a**) WET; (**b**) MQL.

Figure 11 shows a graph of changes in the values of the surface roughness parameters *Rp* and *Rv* depending on the GF supply method used: WET (Figure 11a) and MQL (Figure 11b).

The *Rp* and *Rv* parameters provide good information about the shape of the profile and are useful for abrasion resistance testing. For both methods of supplying GF to the grinding zone, low values of the *Rp* parameter and high values of the *Rv* parameter were obtained. Such parameters characterise surfaces with wide peaks (profile with rounded ridges) and narrow valleys, i.e., surfaces with good abrasion resistance.

**Figure 12:**
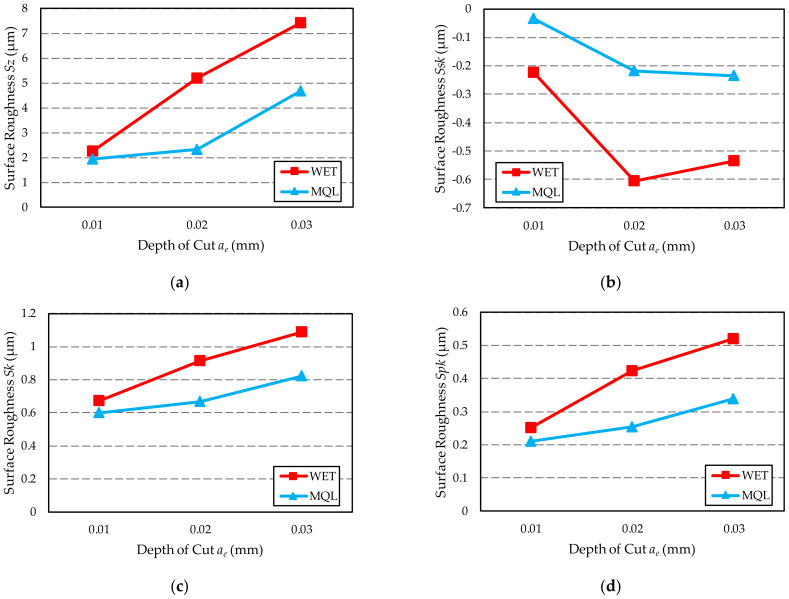
Value of 3D parameters determining the roughness of ground surface with WET and MQL methods: (**a**) *Sz*; (**b**) *Ssk*; (**c**) *Sk*; (**d**) *Spk*.

Table 8 presents the results of measurements of the surface topography described by the 3D amplitude parameters—*Sz*, *Ssk,* and the parameters of the material ratio curve—*Sk* and *Spk*. Figure 12 presents a graphical representation of the obtained results in the form of graphs.

In the case of *Sz* parameter (Figure 12a), which has a high generalisation capacity due to its insensitivity to the influence of individual random peaks and valleys, its lower values were observed, same as for *Rz* parameter, for surfaces ground using the MQL method. In case of the water-oil emulsion fed by the WET method, the obtained surface roughness is 15 to as much as 124% higher when compared to the surface roughness after grinding with GF feeding using the MQL method. The gap between the compared roughness values depends on the depth of grinding. Similarly to the *Rz* parameter, the lowest difference was obtained for the grinding depth *a_e_*_1_ = 0.01 mm.

Negative values of the skewness coefficient *Ssk* (Figure 12b) indicate that surfaces with plateau-like peaks were obtained. At the same time, similar values of *Ssk* indicate that the tested surfaces were free of accidental extreme deviations of the unusual local valleys or peaks. The only exception is the surface ground to a depth of *a_e_*_1_ = 0.01 mm using the MQL method. It must be remembered; however, that skewness is very sensitive to random extreme deviations of the surface, which may significantly influence the *Ssk* value while having no effect at all on the functional properties of the surface.

Comparison of the obtained values of the *Sk* parameter (Figure 12c), which determines the value of roughness of the core and can represent a measure of the effective depth of roughness after the sanding period, showed that *Sk* for the ground surfaces is close to each other. The difference between the minimum and maximum values is 0.491 μm.

The lowest values of the reduced peaks height *Spk* (Figure 12d) were measured for all surfaces ground with GF fed using the MQL method and for the surface ground to a depth of *a_e_*_1_ = 0.01 mm with GF fed using the WET method. This proves the high abrasion resistance of the tested contact surfaces. It should be remembered that the lower the value of this parameter, the better the abrasion resistance of the geometric structure of the surface (ST), which is important in the case of surfaces that work in contact.

**Figure 13:**
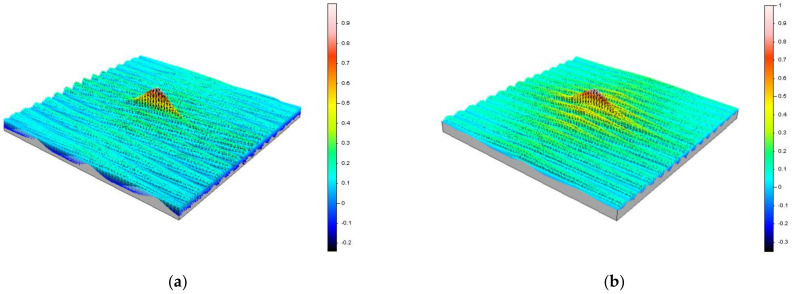
Autocorrelation functions of surfaces obtained by grinding at a grinding depth *a_e_*_1_ = 0.01 mm: (**a**) WET; (**b**) MQL.

Figure 13 shows illustrative images of the autocorrelation function of the measured surfaces. Due to high similarity between the images, the images were limited to the ones obtained for a grinding depth *a_e_*_1_ = 0.01 mm and using both grinding fluid supply methods: the WET method (Figure 13a) and the MQL method (Figure 13b). As it can be seen from the presented images, the autocorrelation functions are exponentially and periodically vanishing, which is characteristic for a random anisotropic surface generally obtained after abrasive machining. Surfaces of this type may have various design purposes, both as contact surfaces (moving and non-moving) and as non-contact surfaces, mainly dynamically or statically loaded.

The anisotropic properties of the tested surfaces are confirmed by the *Str* texture aspect ratio, whose values are listed in Table 9. All the obtained *Str* values are close to 0, which is typical for anisotropic surfaces. It should be noted that for both GF supply methods (WET and MQL), the smallest values of *Str* parameter were obtained for the smallest grinding depth *a_e_*_1_ = 0.01 mm. For the remaining two grinding depths, the values of the *Str* parameter are similar and greater than the smallest value by 86–90% for the WET method and 96–117% for the MQL method. 

**Figure 14:**
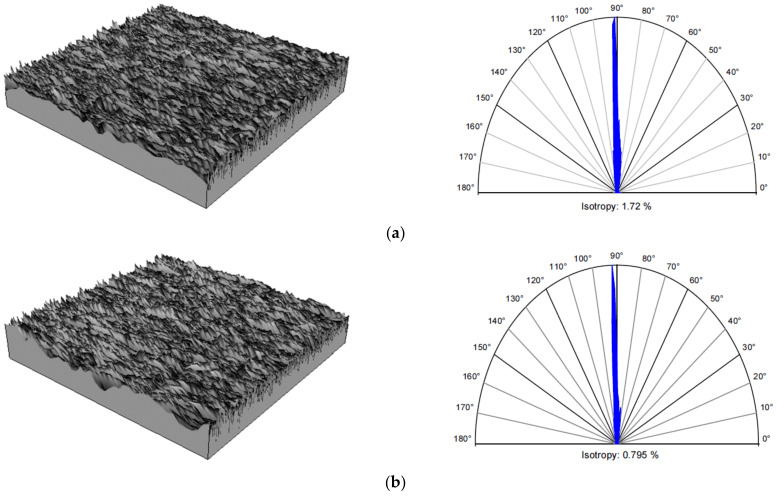
Surface texture and polar graph of the texture directions after grinding with a grinding depth *a_e_*_1_ = 0.01 mm: (**a**) WET; (**b**) MQL.

In addition, the values of the *Str* parameter obtained for the same grinding depths are greater when GF is applied by the WET method. Another confirmation of the anisotropy of the obtained surfaces is the values of isotropy listed in Table 9. It is worth mentioning that isotropy is expressed in percentage: from 0% for the completely anisotropic surface to 100% for the completely isotropic surface. In our case, all isotropy values are close to 0%, not exceeding 3.32%. Figure 14 shows examples of isotropy of surfaces ground to a grinding depth of *a_e_*_1_ = 0.01 mm with grinding fluid supplied using the flood method (Figure 14a) and using the MQL method (Figure 14b). For each case, the surface appearance and the direction indicator showing the main surface texture directions are shown.

## 4. Conclusions

The article describes experimental research aimed at determining the effect of the applied grinding depth *a_e_* on the selected parameters describing the technological condition of the surface layer of flat samples made of 20MnCr5 steel. The samples were vacuum carburised (LPC) in a single-piece flow method and high-pressure gas quenched (HPGQ) in a 4D Quenching chamber. Then the samples were ground with Vortex type, IPA60EH20VTX alumina grinding wheel. The samples were ground at three depths of grinding with grinding fluid supply using either flood method (WET) or with minimum quantity lubrication (MQL) method. The condition of the technological surface layer was described using microhardness and residual stresses, as well as some selected parameters of surface roughness.

Based on the results obtained for the experimental conditions applied, it can be concluded that:

1. For each of the three *a_e_* grinding depths, lower (more favourable) changes in microhardness compared to the microhardness of the material before grinding occur in the surface layer of samples ground with GF fed using MQL method.
2. For both GF supply methods (WET and MQL), the microhardness distribution in the material of samples ground with the smallest grinding depth (0.01 mm) showed no significant differences with respect to the microhardness distribution in the material of these samples after vacuum carburising treatment.
3. The vacuum carburising process carried out by the “single-piece flow” method enables favourable, i.e., compressive distribution of residual stresses to be obtained in the technological surface layer.
4. In general, the grinding process with an alumina grinding wheel causes the residual stresses in the material to deteriorate in comparison with the sample material after vacuum carburising treatment and before grinding.
5. The least unfavourable changes in residual stresses occur during grinding with the lowest grinding depth (0.01 mm), for which residual stresses remain within the range of favourable compressive stresses. For greater grinding depths (≥0.02 mm), the residual stresses move into the unfavourable area of tensile stresses. The above observations apply to both methods (WET and MQL) of supplying GF to the grinding zone.
6. For each of the three *a_e_* grinding depths, lower surface roughness values are obtained after grinding with GF fed with MQL method.
7. For each of the three grinding depths *a_e_*, the obtained values of 3D surface roughness parameters indicate that for both methods of feeding GF into the grinding zone (WET and MQL), random anisotropic surfaces with good abrasion resistance, i.e., with wide peaks and narrow valleys, are obtained.

The conclusions presented above indicate that the MQL method in the studied range of grinding conditions is an alternative to the conventional flood method. Therefore, the amount of GF can be significantly reduced, which is important due to the ecological aspect of the machining. The obtained results are also the basis for further research, taking into account the use of hybrid methods of delivering GF to the grinding zone [42].

The test results and conclusions may be useful for technologists designing manufacturing processes with the use of grinding flat surfaces of samples made of 20MnCr5 steel.

## Figures and Tables

**Table 1:** Thermochemical treatment (TCT) conditions.

Treatment	Process Parameters	Value
Vacuum carburising	Temperature	920 °C
Quenching	Medium	Nitrogen
	Pressure	7 bar (0.7 MPa)
	Precooling	850 °C
Tempering	Temperature	190 °C
	Time	180 min

**Table 2:** Chemical composition of the 20MnCr5 steel.

Content of Elements (wt.%)							
C	Mn	Cr	Si	Ni	Cu	P	S
0.20	1.35	1.12	0.18	0.11	0.10	0.015	0.022

**Table 3:** Grinding conditions.

Grinding mode	Single-pass longitudinal circumferential surface grinding
Grinding machine	Flat-surface grinder SPD-30B (Jotes Inc., Lodz, Poland)
Workpiece material	20MnCr5, carburized and hardened with 820 ± 10 HV
Grinding wheel	IPA60EH20VTX (Vortex type)
Grinding wheel rotational speed	*n_s_* = 1650 rpm
Grinding wheel peripheral speed	*v_s_* = 30.2 m/s
Workpiece peripheral speed	*v_w_* = 18 m/min
Working engagement (machining allowance)	*a_e_*_1_ = 0.01 mm *a_e_*_2_ = 0.02 mm *a_e_*_3_ = 0.03 mm
Dresser	Single grain diamond dresser type M1020
Dresser weight	*Q_d_* = 2.0 kt
Grinding wheel peripheral speed while dressing	*v_sd_* = 10 m/s
Dressing allowance	*a_d_* = 0.02 mm
Axial table feed speed while dressing	*v_fd_* = 5.0 mm/min
Number of dressing passes	*i_d_* = 4
Environments	WET—conventional fluid MQL—minimum quantity lubrication
Conventional grinding fluid (GF)	Emulgol ES-12 in a 5% concentration
Conventional GF flow rate	*Q_WET_* = 4 L/min
MQL system	Ecolubric MQL Booster—oil-mist generator with single external nozzle
MQL fluid	Ecolubric E200L—cold-pressed rapeseed oil without additives
MQL flow rate	*Q_MQL_* = 100 mL/h
MQL supply air pressure	*P* = 0.6 MPa

**Table 4:** Characteristics of Ecolubric E200L rapeseed oil applied in the research.

Properties	Description
Chemical description	A fraction of natural triglycerides, easily biodegradable substances
Density at 0 °C	0.9273 g/cm^3^
Dynamic viscosity at 0 °C	2.881 N s/m^2^
Ignition point	365 °C
Flash point	325 °C
Partition coefficient	<3%
Health hazard	Not hazard to human health

**Table 5:** Variable grinding conditions applied in the research.

Number of Samples	Grinding Depth *a_e_* (mm)	Method of Coolant-Lubricant Supply
1-W	0.01	WET
1-M		MQL
2-W	0.02	WET
2-M		MQL
3-W	0.03	WET
3-M		MQL

**Table 6:** Surface roughness measuring conditions.

Type of Profilometer	Hommel Tester T8000 (Hommelwerke GmbH, Schwenningen, Germany)
Stylus type	TKU 300
Tracing length	*lt* = 4.8 mm
Evaluation length	*ln* = 4.0 mm
Sampling length	*lr* = 0.8 mm
Evaluation width (3D measurements)	*l* = 5 mm
Number of stylus passes (3D measurements)	51
Distance between stylus tracks (3D measurements)	0.1 mm
Stylus tip radius	*r_tip_* = 2 μm
Stylus tip angle	90°
Tracing speed	*v_t_* = 0.05 mm/s
Long-wave profile filter (cutoff)	*λ_c_* = 0.8 mm
Measuring range	±80 μm

**Table 7:** Surface roughness 2D parameters.

Method of Coolant-Lubricant Supply	Grinding Depth *a_e_* (mm)	Surface Roughness (μm)		
		*Rp*	*Rv*	*Rz*
WET	0.01	0.92	1.09	2.00
	0.02	1.36	1.52	2.88
	0.03	1.55	1.82	3.33
MQL	0.01	0.76	0.92	1.67
	0.02	0.88	1.09	1.96
	0.03	1.10	1.52	2.62

**Table 8:** Surface roughness 3D parameters.

Method of Coolant-Lubricant Supply	Grinding Depth *a_e_* (mm)	Surface Roughness (μm)			
		*Sz*	*Ssk*	*Sk*	*Spk*
WET	0.01	2.25	−0.223	0.673	0.252
	0.02	5.20	−0.606	0.916	0.424
	0.03	7.42	−0.534	1.090	0.520
MQL	0.01	1.95	−0.034	0.599	0.211
	0.02	2.32	−0.218	0.669	0.254
	0.03	4.69	−0.234	0.820	0.340

**Table 9:** Surface roughness 3D parameters.

Method of Coolant-Lubricant Supply	Grinding Depth *a_e_* (mm)	Texture Aspect Ratio	Isotropy (%)
		*Str* (-)	
WET	0.01	0.0172	1.72
	0.02	0.0320	3.20
	0.03	0.0332	3.32
MQL	0.01	0.0079	0.80
	0.02	0.0172	1.72
	0.03	0.0155	1.55

## Data Availability

All data generated or analysed during this study are included in this article.

## Nomenclature

ECD	Effective case depth
GF	Grinding fluid
HPGQ	High-pressure gas quenching
HV	Hardness in the Vickers scale
LPC	Low pressure carburising
MQL	Minimum quantity lubrication
PSD	Power spectral density
TCT	Thermochemical treatment
WET	Flood method using water emulsion as coolant
*a_e_*	Working engagement (depth of cut), mm
*a_d_*	Dressing allowance, mm
*i_d_*	Number of dressing passes
*l*	Evaluation width, mm
*lt*	Tracing length, mm
*ln*	Evaluation length, mm
*lr*	Sampling length, mm
*n_s_*	Grinding wheel rotational speed, rpm
*P*	Supply air pressure (MQL method), MPa
*r_tip_*	Stylus tip radius, μm
*Rp*	Value of the highest peak of the profile, μm
*Rv*	Value of depth of the lowest valley of the profile, μm
*Rz*	Maximum height of the roughness profile, μm
*Sk*	Core roughness depth, μm
*Spk*	Reduced peak height, μm
*Ssk*	Skewness, μm
*Str*	Texture direction
*Sz*	Maximum height of surface, μm
*Q_d_*	Dresser weight, kt
*Q_MQL_*	MQL flow rate, mL/h
*Q_WET_*	Conventional GF flow rate, L/min
*v_fd_*	Axial table feed speed during dressing, mm/min
*v_s_*	Grinding wheel peripheral speed, m/s
*v_sd_*	Grinding wheel peripheral speed during dressing, m/s
*v_t_*	Tracing speed, mm/s
*v_w_*	Workpiece peripheral speed, m/min
*λc*	Long-wave profile filter, mm
